# Supplementary material for: Fucoidan Isolated from Saccharina japonica Inhibits LPS-Induced Inflammation in Macrophages via Blocking NF-κB, MAPK and JAK-STAT Pathways
Source: Mar Drugs. 2020 Jun 24;18(6):328. doi: 10.3390/md18060328 (PMC7345355; doi:10.3390/md18060328)
Supplement: Supplementary File 1 [file marinedrugs-18-00328-s001.pdf]

Supporting Information

Fucoidan isolated from *Saccharina japonica* inhibit LPS-induced inflammation in macrophages

Jing Ye[1,2*], Donghui Chen[1], Zhicheng Ye[1], Yayan Huang[1], Na Zhang[1,2], Edmund M. K. Lui[3], Changhu Xue[4], and Meitian Xiao[1,2]

[1] College of Chemical Engineering, Huaqiao University, Xiamen 361021, China

[2] Xiamen Engineering and Technological Research Center for Comprehensive Utilization of Marine Biological Resources, Xiamen 361021, China

[3] Physiology and Pharmacology, Western University, London Ontario N6A 5B9, Canada

[4] College of Food Science and Engineering, Ocean University of China, Qingdao 266003, China

*** Corresponding author:**

Jing Ye

College of Chemical Engineering, Huaqiao University, Xiamen 361021, Fujian, China

E-mail: yejenny@hqu.edu.cn, Tel./fax: +86-592-6162300

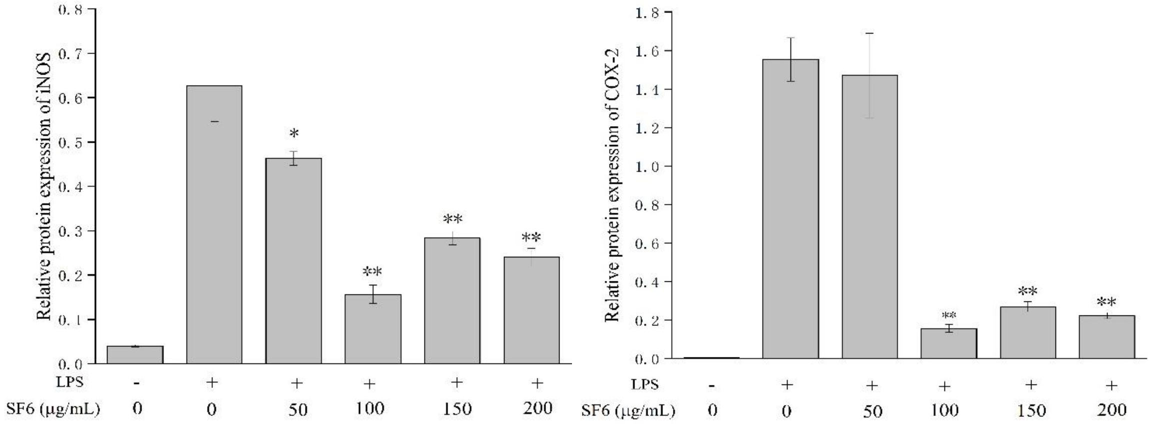

Figure S1 Effects of SF6 on the relative protein expression of iNOS and COX-2 in LPS-stimulated RAW264.7 cells.

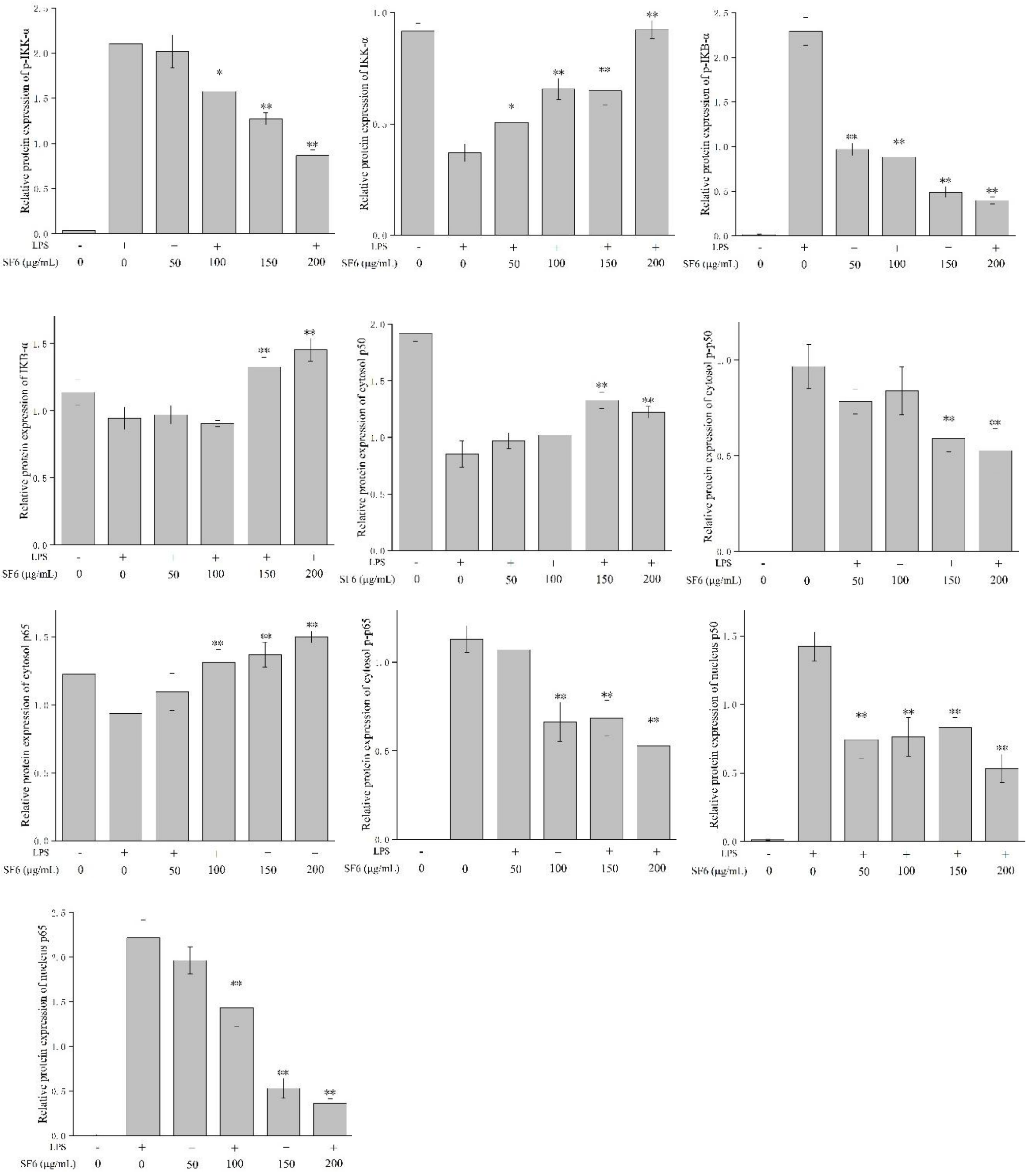

Figure S2 Effects of SF6 on the relative protein expression of the NF-κB signaling pathway in LPS-stimulated RAW264.7 macrophages.

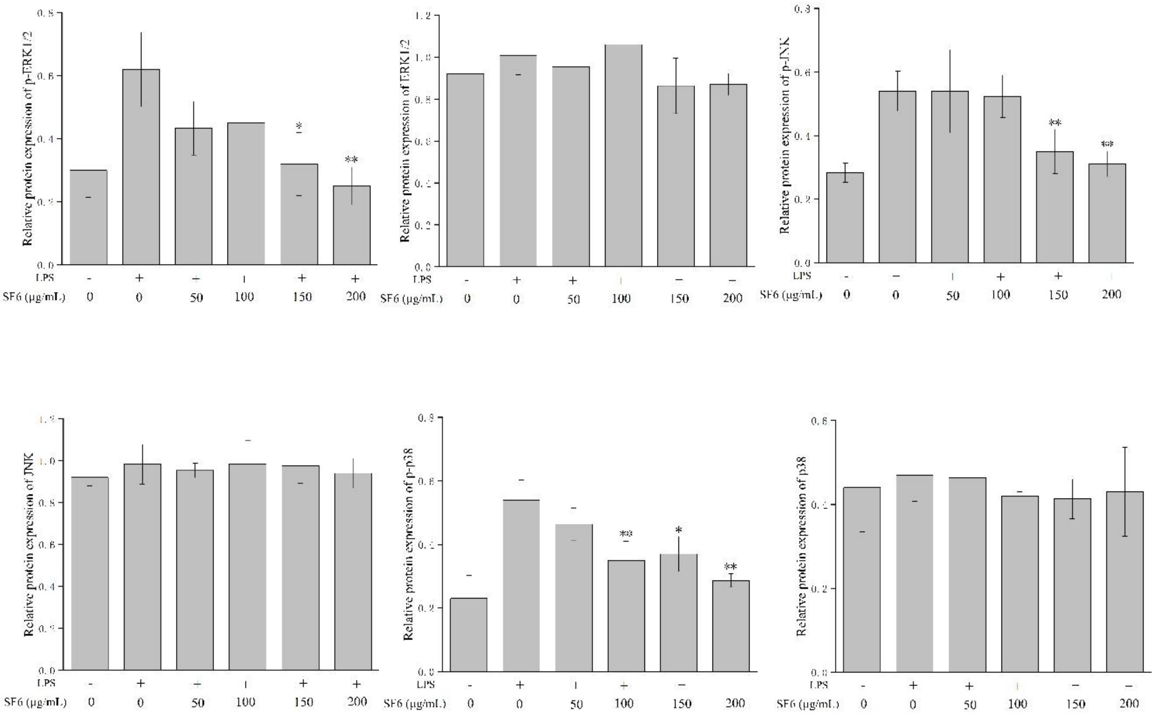

Figure S3 Effects of SF6 on the relative protein expression of the NF-κB signaling pathway in LPS-stimulated RAW264.7 macrophages.

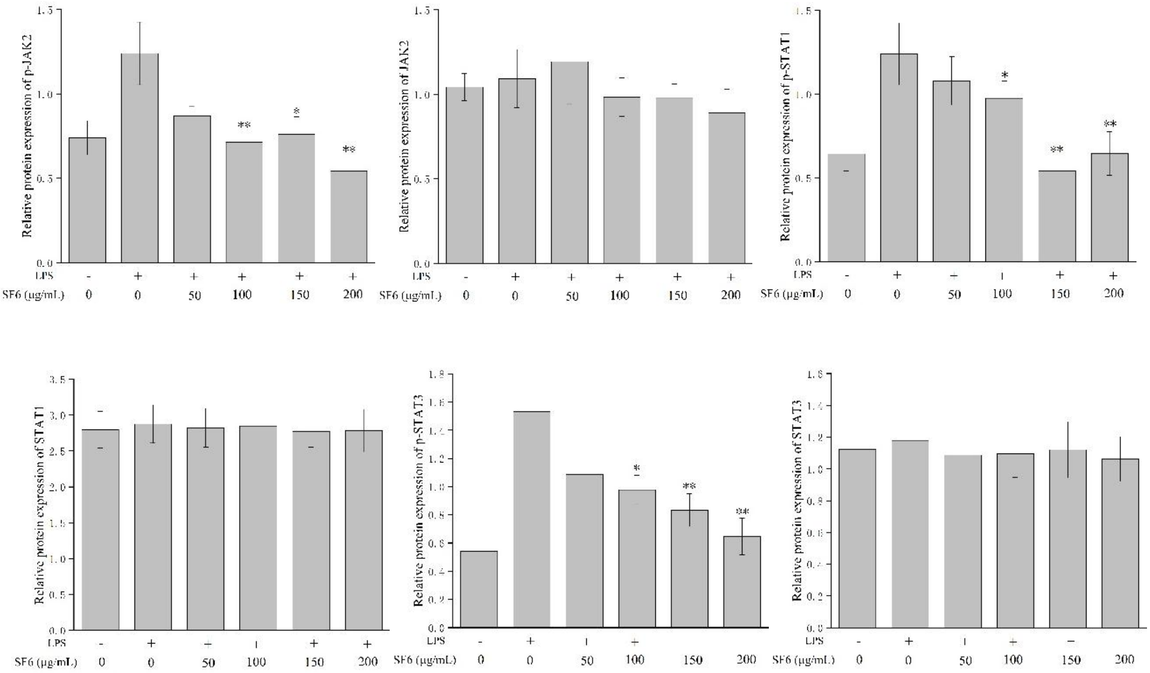

Figure S4 SF6 Effects of SF6 on the relative protein expression of the JAK2-STAT1/3 signaling pathway in LPS-stimulated RAW264.7 macrophages.
